# Analyzing the expression and clinical significance of CENPE in gastric cancer

**DOI:** 10.1186/s12920-024-01887-7

**Published:** 2024-05-03

**Authors:** Jing Wang, Xiaofei Li, Xihui Qiang, Xueqing Yin, Lianyi Guo

**Affiliations:** Department of Gastroenterology, The First Affiliated Hospital of Jinzhou Medical University, Jinzhou, 121001 China

**Keywords:** CENPE, Gastric cancer, Bioinformatics

## Abstract

**Background:**

Gastric cancer (GC) is a prevalent type of malignant gastrointestinal tumor. Many studies have shown that CENPE acts as an oncogene in some cancers. However, its expression level and clinical value in GC are not clear.

**Methods:**

Obtaining clinical data information on gastric adenocarcinoma from TCGA and GEO databases. The gene expression profiling interaction analysis (GEPIA) was used to evaluate the relationship between prognosis and CENPE expression in gastric cancer patients. Utilizing the UALCAN platform, the correlation between CENPE expression and clinical parameters was examined. Functions and signaling pathways of CENPE were analyzed using the Gene Ontology (GO), the Kyoto Encyclopedia of Genes and Genomes (KEGG), and Gene Set Enrichment Analysis (GSEA). The association between immunological infiltrating cells and CENPE expression was examined using TIMER2.0. Validation was performed by real-time quantitative PCR (qPT-PCR) and immunohistochemical analysis.

**Results:**

According to the analysis of the GEPIA database, the expression of CENPE is increased in gastric cancer tissues compared to normal tissues. It was also found to have an important relationship with the prognosis of the patient (*p*<0.05). The prognosis was worse and overall survival was lower in individuals with increased expression of CENPE. In line with the findings of the GEPIA, real-time fluorescence quantitative PCR (qPT-PCR) confirmed that CENPE was overexpressed in gastric cancer cells. Furthermore, It was discovered that H. pylori infection status and tumor grade were related to CENPE expression. Enrichment analysis revealed that CENPE expression was linked to multiple biological functions and tumor-associated pathways. CENPE expression also correlated with immune-infiltrating cells in the gastric cancer microenvironment and was positively connected to NK cells and mast cells. According to immunohistochemical examination, paracancerous tissues had minimal expression of CENPE, but gastric cancer showed significant expression of the protein.

**Conclusions:**

According to our findings, CENPE is substantially expressed in GC and may perhaps contribute to its growth. CENPE might be a target for gastric cancer therapy and a predictor of a bad prognosis.

## Introduction

Gastric cancer (GC), which positions fourth in death and fifth in incidence worldwide, is one of the most deadly diseases, with approximately 7,690,000 new diagnoses in 2020 [1]. It affects males six times more than women [1]. Despite significant progress in research on GC, the majority of cases are still identified at the late stages of the disease due to multiple risk factors and molecular mechanisms that are not yet fully understood. Radiation, chemotherapy, surgery, and targeted therapy are common forms of treatment. GC remains a major challenge, posing a serious threat to people's health and lives. Therefore, it is essential to explore new biological markers to provide personalized treatment and potential therapeutic targets for gastric cancer treatment.

CENPE is a kind of microtubule kinesin that is essential for mitosis and helps to regulate the interactions between kinetochore and spindle [2]. Its expression is upregulated in the G2 phase, peaking in the M phase, and it uses microtubule movement to separate chromosomes at the equatorial plate [3]. Abnormal deletion of this protein can result in incorrect chromosomal alignment at mitosis, ultimately leading to the halt of the M stage [2]. CENPE expression is closely associated with tumorigenesis and development. Studies have shown that increased CENPE expression is linked to clinical stage and overall survival in glioblastoma, and inhibiting its expression leads to the inhibition of glioblastoma cell proliferation [4]. In addition, CENPE alteration ultimately inhibits in vitro expansion of medulloblastoma cells by disrupting mitosis and DNA damage [5]. In non-small cell lung cancer, CENPE overexpression is significantly correlated with prognosis [6]. High CENPE expression correlates with methylation in esophageal adenocarcinoma [7]. while in ovarian cancer, it interacts with kinesin family member C1 (KICF1) to boost the development, migration, and epithelial-mesenchymal switch of ovarian cancer [8]. However, CENPE 's functions in the development of GC and its related mechanisms remain unknown.

Therefore, in this study, samples related to gastric cancer were obtained from TCGA and GEO databases, and the differential gene CENPE was identified through a joint screening process. Multiple public databases were then used to explore the expression, prognosis, biological function, and related signal pathways of CENPE in gastric cancer. The findings were further verified through real-time fluorescence quantitative PCR and immunohistochemical staining. The purpose of this study was to explore the effect and clinical significance of CENPE in gastric cancer.

## Methods

### Individuals and samples of tissue

Thirty cases of GC tissues and adjacent paracancerous tissues were collected for this research, and specimens were collected from December 2021 to December 2023 in the First Affiliated Hospital of Jinzhou Medical University. All subjects had not received chemotherapy, radiotherapy, or other related tumor treatments, and the patients and their families were fully informed and obtained informed consent. The obtained fresh tissue was frozen with liquid nitrogen and then frozen in an ultra-low temperature refrigerator at -80℃ in the laboratory for use. The First Affiliated Hospital of Jinzhou Medical University's ethical council gave its approval to the project. (KYLL 2023119 ).

### Screening of differential genes

From the TCGA database (https://portal.gdc.cancer.gov/), the expression of gene data and clinical data pertaining to gastric cancer were obtained, 375 gastric adenocarcinoma specimens and 32 normal samples were obtained. GSE118916 and GSE2685 were downloaded from the GEO database (https://www.ncbi.nlm.nih.gov/geo/), which has 60 samples, including 37 tumor samples and 23 normal samples. After sorting the data, the limma package from the R language was used to compare gastric cancer and normal samples to find differentially expressed genes.

### Cell culture

Normal cell lines for the stomach epithelium (GSE-1) and Gastric cancer cells (BGC-823, AGS, MKN-45) were purchased from Wuhan Cell Bank, and have been raised in RPMI 1640 media (Beijing Solarbio Science & Technology Co., Ltd.) with a fetal bovine serum content of 10% (Sijiqing, Zhejiang Tianhang Biotechnology Co., LTD) and incubated at 37°C with 5% CO2.

### Examination of the prognosis and expression of CENPE in GC

We used the Boxplot module in the GEPIA(http://gepia.cancer-pku.cn/) database to analyze the expression of CENPE in gastric cancer, and set |log2FC| Cutoff ≤1 and p-value Cutoff ≤ 0.01.The prognosis of CENPE for patients with gastric cancer will be analyzed in the Survival Plots module.

### Relationship between CENPE expression and various clinical parameters

UALCAN (https://ualcan.path.uab.edu/index.html) is a comprehensive web-based resource for canceromics data analysis, based primarily on relevant cancer information in TCGA. The correlation between CENPE and gender, tumor grade, individual cancer stage, and H. pylori infection status was analyzed by the Expression module.

### Analysis of GO and KEGG

GO and KEGG were used to analyze the biological roles of CENPE in gastric cancer. In the GO analysis, from molecular function (MF), Cell structure (CC), and biological process (BP) Three aspects were analyzed. KEGG focuses on the metabolic pathways in the organism. We performed the analysis by using the R software clusterProfiler package and visualized the results.

### GSEA

GSEA software uses pre-defined gene sets arranged according to differential expression levels for gene set enrichment analysis. We used GSEA to evaluate the relationship between CENPE expression and signaling pathways.

### Analysis of immune infiltration

To further understand the immune microenvironment of the tumor, we analyzed the expression levels of 22 kinds of immune cells based on the CIBERSORT algorithm. The relationship between immune infiltrating cells and CENPE expression was analyzed using the TIMER2.0 database (http://timer.cistrome.org/). It is a tumor immunoassay database with three main sections: immunity, exploration, and evaluation, which comprehensively analyzes tumor immune cell infiltration and provides visualization.

### Real-time fluorescence quantitative PCR analysis

As directed by the manufacturer, we extracted total RNA from the cell lines using the RNA easy^TM^ Animal RNA Isolation Kit (Beyotime, China) and measured the RNA concentration using a spectrophotometer. The cDNA was transcribed according to the All-in-one RT SuperMix Perfect for qPCR instructions(Vazyme Biotech Co., Ltd). The cDNA was then subjected to real-time fluorescence quantitative PCR using SYBR qPCR Master Mix (Vazyme Biotech Co., Ltd). PCR thermal cycling instrument (Bio-techne, Germany) was used, and the setting conditions were:95℃, 30min, 95℃, 10s, 60℃, 30min, and 40 cycles. The experiment was performed using a Bio-Rad PCR instrument (Bio-Rad, USA). Detection of the relative expression of CENPE was performed by the 2-ΔΔCT approach. GAPDH serves as an inner control. The following were primer sequences:

- CENPE (F): 5'-ACTCAAGGAAAGCCTGCAAGA-3'
- CENPE (R): 5'-GGTTCTGTCGGTCCTGCTTT-3'
- GAPDH (F): 5'-AATGGGCAGCCGTTAGGAAA-3'
- GAPDH (R): 5'-GCCCAATACGACCAAATCAGAG-3'

### Immunohistochemical analysis

The paraffin embedded GC tissues were cut into slices that were 5um thick. These sections were then dewaxed with xylene and hydrated in ethanol of varying concentrations. Antigen repair was performed using citrate buffer. The tissue sections were then closed with 5% goat serum (Beijing Solarbio Science & Technology Co., Ltd.) and left to stand for 20 min at 37℃. Tissue slices were incubated with the original antibody (CST, CatNo.14977S) for a whole night at 4°C, after which the sections were placed in Goat anti-rabbit antibody for 35min at37℃(Abcam, 7602). Finally, the color was developed with DAB, washed, and then restained with hematoxylin. Two experienced pathologists performed a double-blind reading of the sections. Sections were graded based on the proportion of stained cellular samples, with <5% as 0 points, <25% as 1 point, 26%-50% as 2 points, 51%-75% as 3 points, and >75% as 4 points. Stained cells were also graded based on the intensity of the staining, with colorless graded as 0, light yellow graded as 1, yellowish brown graded as 2, and sepia graded as 3. The positive grade was obtained by multiplying both scores, with the possible grades being 0 negative (-), 1-4 weak (+), 5-8 moderate (+++), and 9-12 strong (+++).

### Statistical analysis

R(4.2.0) Language was utilized to process data, GraphPad Prism9 software was used for statistical plotting, ImageJ software was used to analyze immunohistochemical staining results, and* t*-test was applied for analyzing data that were normally distributed. *P* < 0.05 was considered statistically significant.

## Results

### Screening of differential genes

**Fig. 1:**
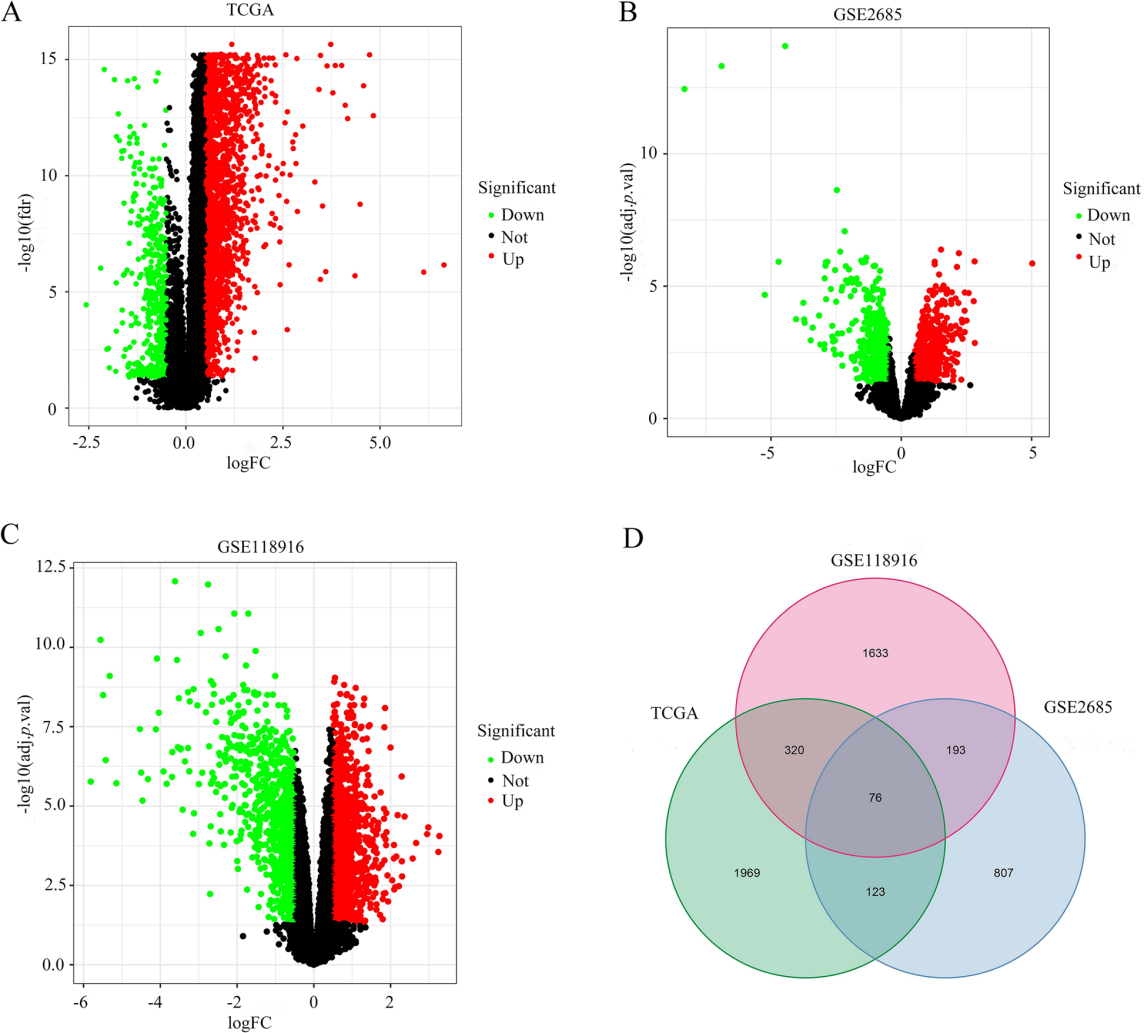
**A-C** Volcano plots of differentially expressed genes in TCGA, GSE2685, and GSE118916 databases in gastric cancer tissues and normal tissues. **D** Wayne plots of differentially expressed genes

Relevant gene expression information was obtained from the TCGA and GEO database (GSE2685, GSE118916), and the limma software package was utilized to do differential analysis to identify the differentially expressed genes (DEGs) with tumor and normal samples., with filtering conditions of *p*< 0.05, |logFC| ≥ 0.5. Volcano plots showed that the lower-regulated genes were green, while the up-regulated genes were red (Fig. 1A-C). The differentially expressed genes of TCGA and GEO were taken as intersections, and finally, 76 DEGs were obtained (Fig. 1D). After screening, it was discovered that CENPE was differentially expressed in GC and normal samples.

### Expression and prognostic analysis of CENPE in gastric cancer

**Fig. 2:**
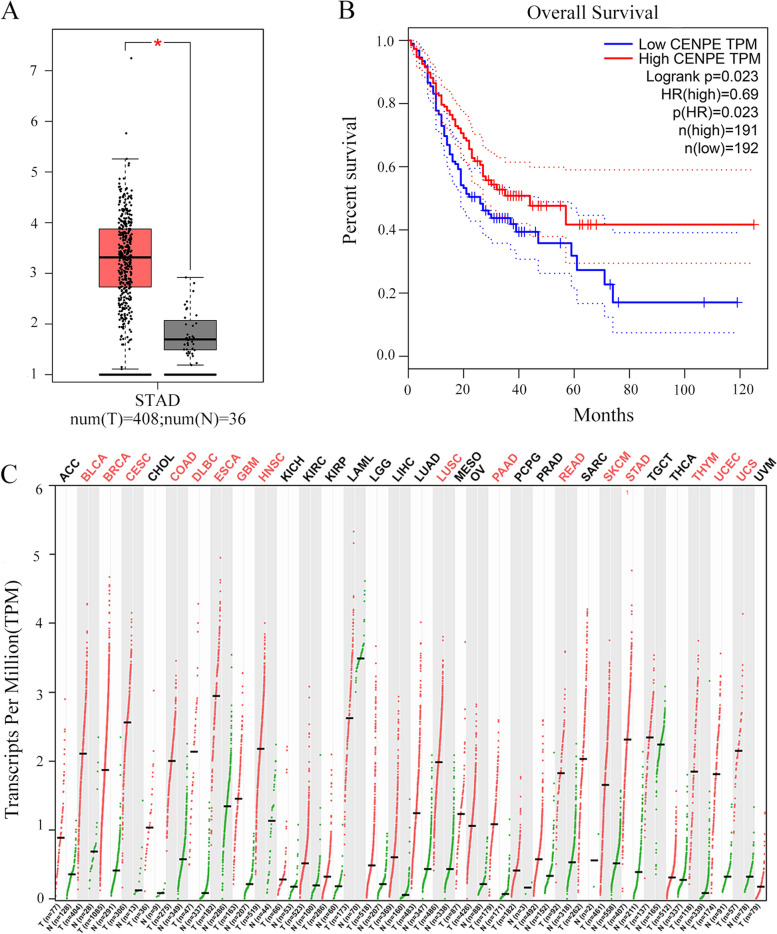
**A** Expression levels of CENPE between gastric cancer tissues and normal tissues. **B** Comparison of the difference in overall survival between gastric cancer patients with high expression of CENPE and gastric cancer patients with low expression of CENPE.** C** CENPE expression in multiple human cancers

First, we evaluated the expression of CENPE in GC tissues and normal tissues by using the GEPIA online analysis tool. According to the findings, GC tissues had higher levels of CENPE expression than normal tissues (Fig. 2A). Next, survival analysis was performed, and as shown (Fig. 2B), In GC individuals, CENPE expression and overall survival (OS) showed a strong correlation. It was shown that patients with gastric cancer who expressed high levels of CENPE had a lower overall survival (OS) compared to those who had low levels of CENPE. *P*< 0.05 was statistically significant. Therefore, high expression of CENPE in GC is associated with the prognosis of patients.

We also analyzed CENPE expression in common tumors. The results showed that compared with normal, CENPE was expressed in bladder uroepithelial carcinoma (BLAC)), breast invasive carcinoma (BRCA), rectal adenocarcinoma (READ), colon adenocarcinoma (COAD), lung squamous cell carcinoma (LUSC), head and neck squamous cell carcinoma (HNSC), esophageal carcinoma (ESCA), stomach adenocarcinoma (STAD) and other tumors with significantly increased expression. Green represents normal tissues and red represents tumor tissues (Fig. 2C).

### Validation of CENPE expression level in gastric cancer by RT-PCR

**Fig. 3:**
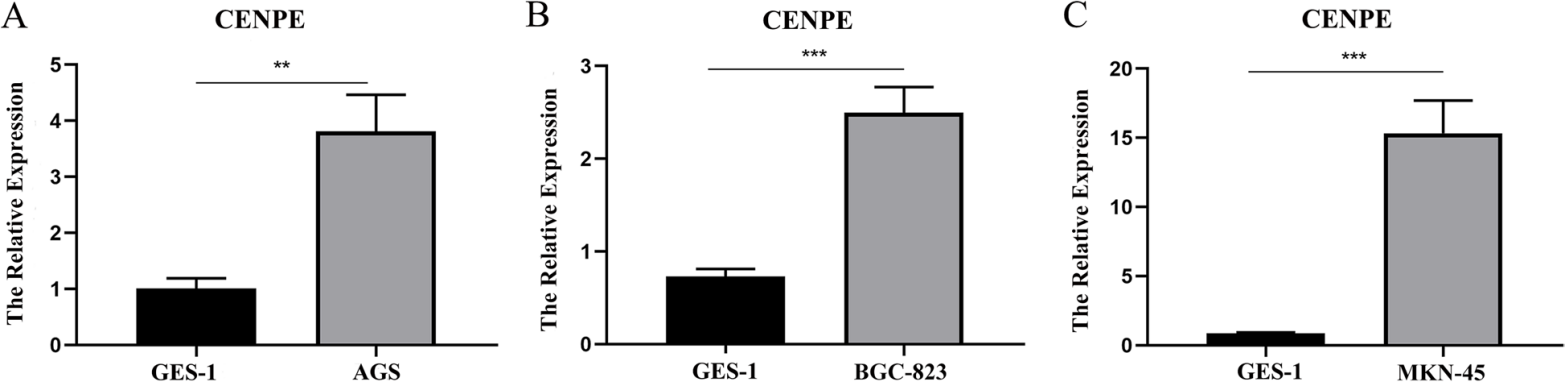
**A** CENPE expression in AGS cells and GSE-1 cells. **B** CENPE expression in BGC-823 cells and GSE-1 cells. **C** CENPE expression in MKN-45 cells and GSE-1 cells

To further verify that the expression of CENPE in GC was higher than in normal cells, We investigated the degree of CENPE expression mRNA in normal gastric epithelial cell line (GSE-1) and GC cell lines (BGC-823, AGS, MKN-45) by the experiment. It was revealed that (Fig. 3A-C) CENPE expression in AGS, BGC-823, and MKN-45 were also increased than GSE-1. Taken together, it can be seen that CENPE mRNA expression was upregulated in GC.

### Detection of CENPE expression in paracancerous and gastric cancer tissues by IHC

**Fig. 4:**
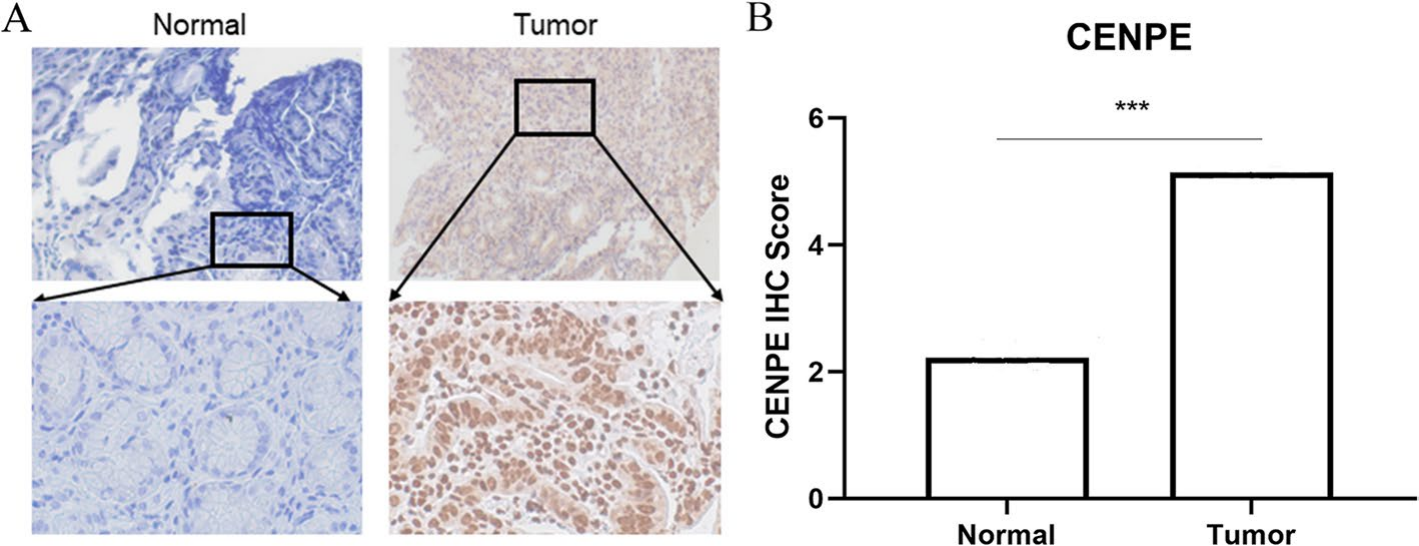
**A** shows the IHC staining of CNEPE in paraneoplastic tissues and gastric cancer tissues at 100X and 200X magnification, respectively.** B** Comparison of immunohistochemical scores

We collected 30 samples of paracancerous and GC, respectively. IHC was applied to study the expression of CENPE. The results of the study showed (Fig. 4A-B) that the expression of CENPE in GC was higher than in paracancerous tissues. That is, in GC, CENEP was strongly expressed. (*P* < 0.001).

### Correlation of CENPE expression with different clinical parameters

**Fig. 5:**
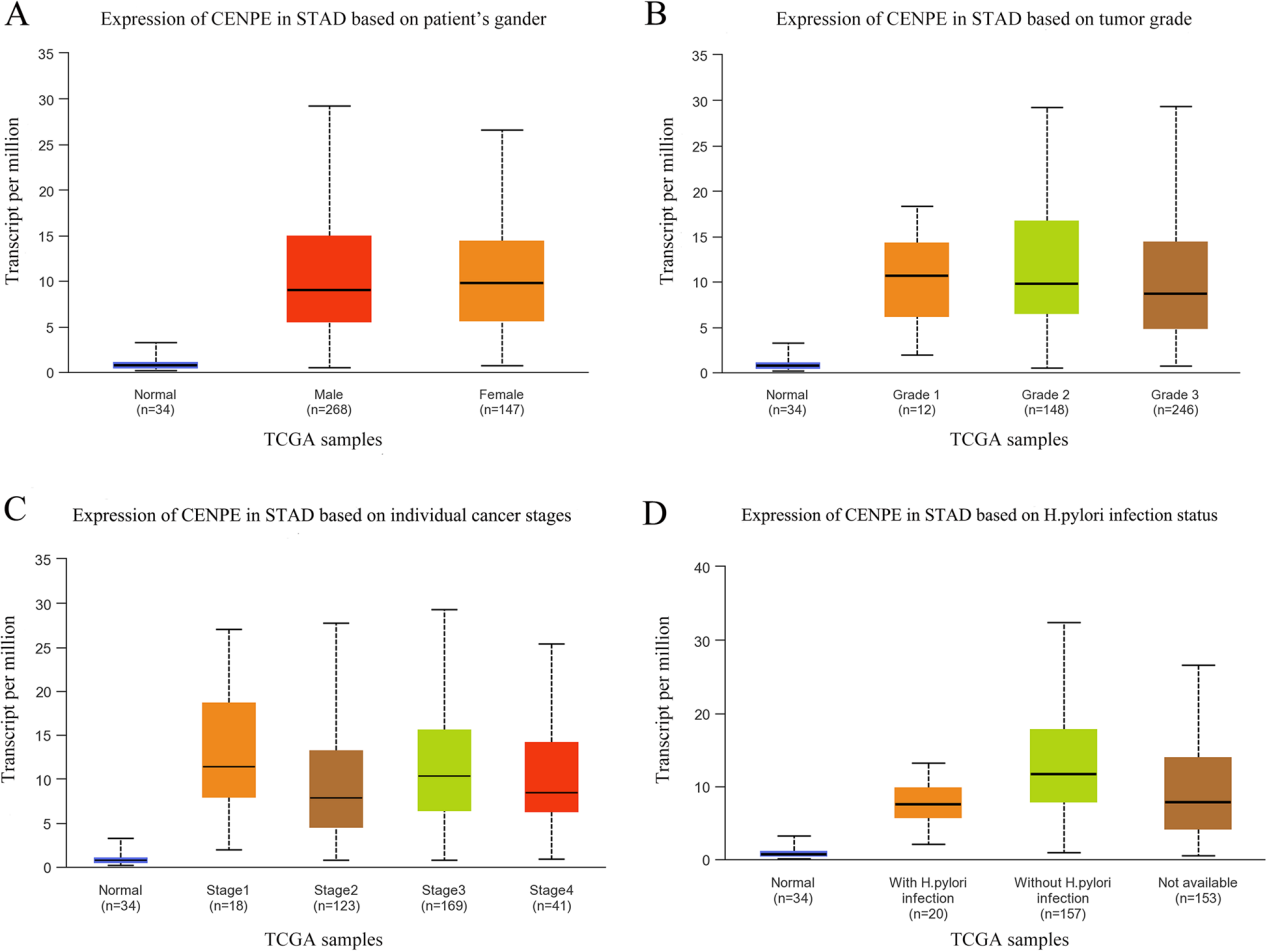
**A-D** Analysis of the relationship between CENPE expression in gastric cancer and clinical parameters (gender, tumor grade, individual cancer stage, H. pylori infection status)

The correlation of CENPE expression with different clinical parameters was examined using the UALCAN online data tool. The results show that, in terms of gender, CENPE expression was higher in GC patients than in normal controls, but there was no apparent distinction between males and females (Fig. 5A). Based on tumor grade, CENPE expression in grades 1 and 3 was a notable variation (Fig. 5B) (*P* < 0.05). In addition, on individual cancer stages, when compared to normal, CENPE expression was considerably higher in stages 1, 2, 3, and 4 (Fig. 5C). However, CENPE expression was not significantly different between stages 1, 2, 3 and 4. Based on H. pylori infection status, CENPE expression differed between infected and uninfected patients (*p* < 0.05) (Fig. 5D).

### GO and KEGG analysis of DEGs

**Fig. 6:**
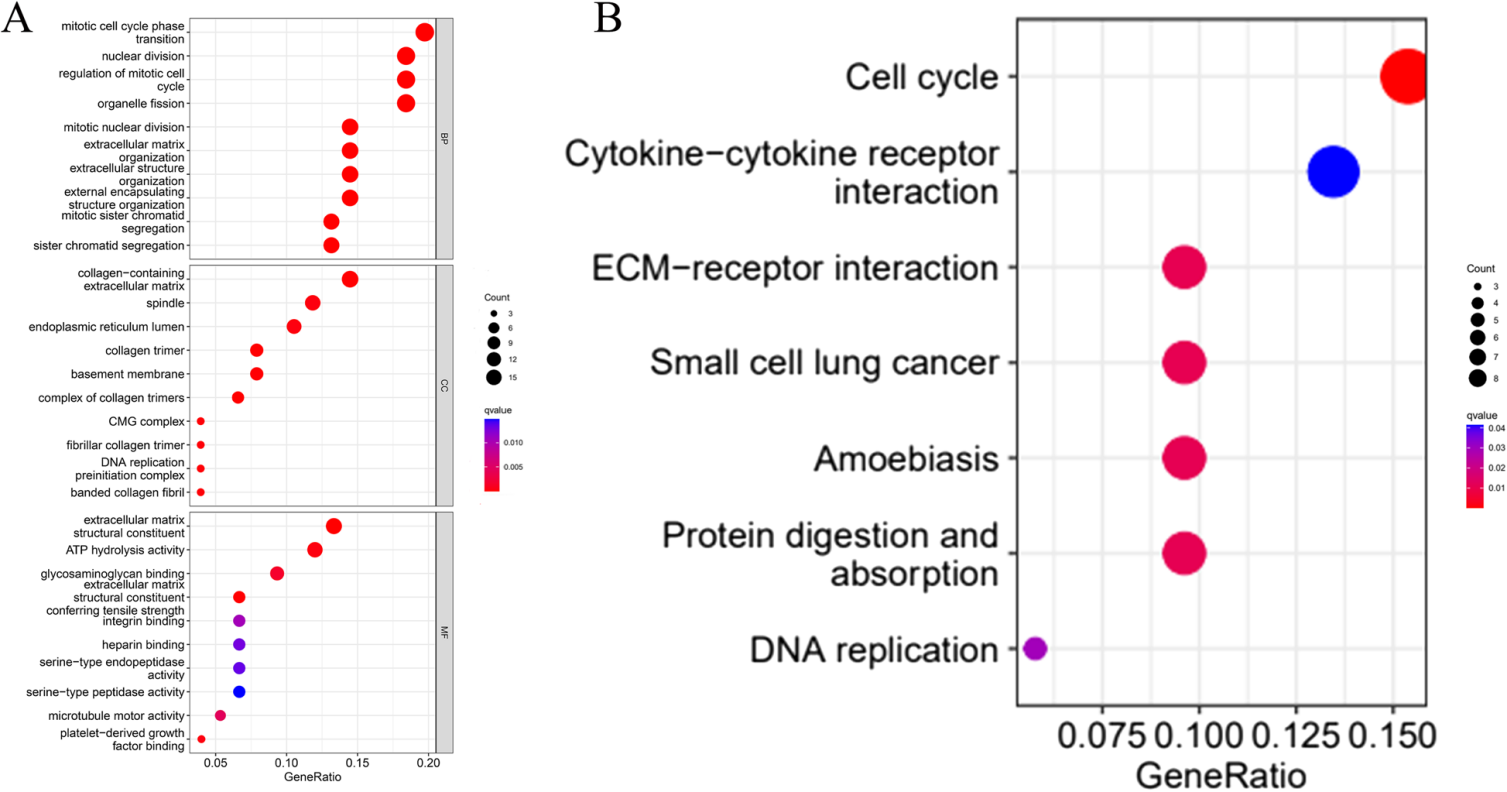
**A** GO enrichment analysis of differentially expressed genes (DEGs).** B** KEGG enrichment analysis of differentially expressed genes (DEGs)

To investigate the signaling pathways and biological functions of CENPE, we mapped the possible biological functions of the DEGs overlapping the Wayne diagrams and analyzed them by utilizing the clusterProfiler software package to analyze GO in terms of the BP, the CC, and the MF in which the genes are involved, followed by KEGG analysis. GO analysis shows (Fig. 6A) that in the BP, CENPE was mainly associated with mitotic cell cycle phase transition, nuclear division, mitotic cycle regulation, and organelle division. For cellular composition, CENPE was mainly associated with extracellular matrix-containing collagen, spindle, and endoplasmic reticulum lumen. For molecular function, CENPE was mainly associated with extracellular matrix structural composition, ATP hydrolyzing activity, and glycosaminoglycan binding to the extracellular matrix. In addition, KEGG analysis shows that DEGs had a relationship with the cell cycle, small-cell lung cancer, small-cell lung cancer, amoebiasis, and digestion and absorption of the protein (Fig. 6B).

### GSEA identifies CENPE-connected signaling pathways

**Fig. 7:**
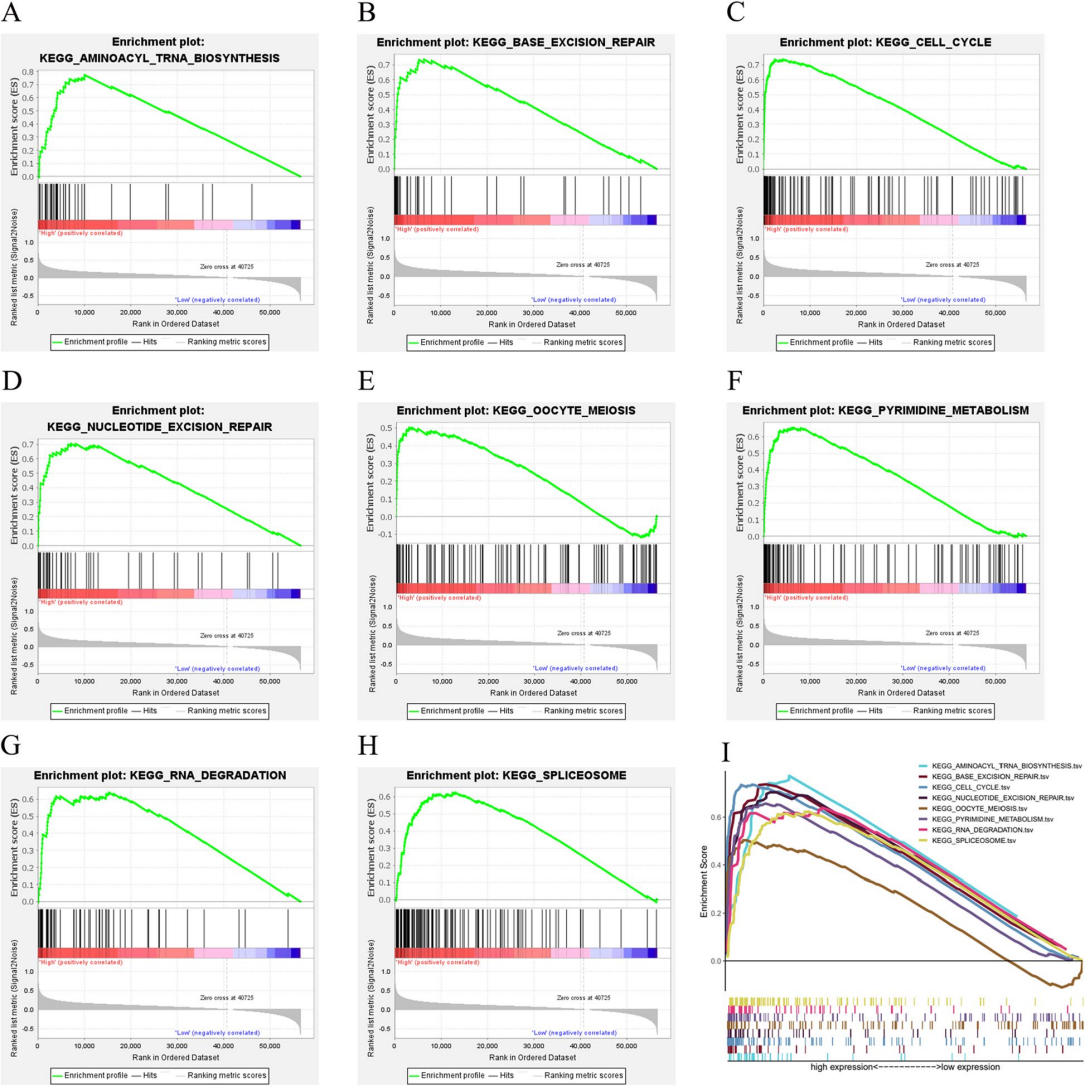
CENPE enrichment map based on GSEA. **A** Aminoacyl TRNA biosynthesis. **B** Base excision RNA repair. **C** Cell cycle. d: Nucleotide excision repair. **E** Oocyte meiosis. **F** Pyrimidine metabolism. **G** RNA degradation. **H** RNA spliceosome. **I** All eight significantly enriched signaling pathways.

We used GSEA to explore the mechanism of CENPE in gastric cancer. After multiple differential sequencing of the genes in the gene set to be studied, the results showed that CENPE was dramatically distinct between the low expression group and the high. expression group. Compared with the low expression group, the major pathways involved in the high expression group of CENPE, which included base excision repair, amino acid TRNA biosynthesis, cell cycle, nucleic acid excision repair, oocyte meiosis, pyrimidine metabolism, RNA degradation, and spliceosome (Fig. 7A-I).

### CENPE expression and immune cell infiltration

**Fig. 8:**
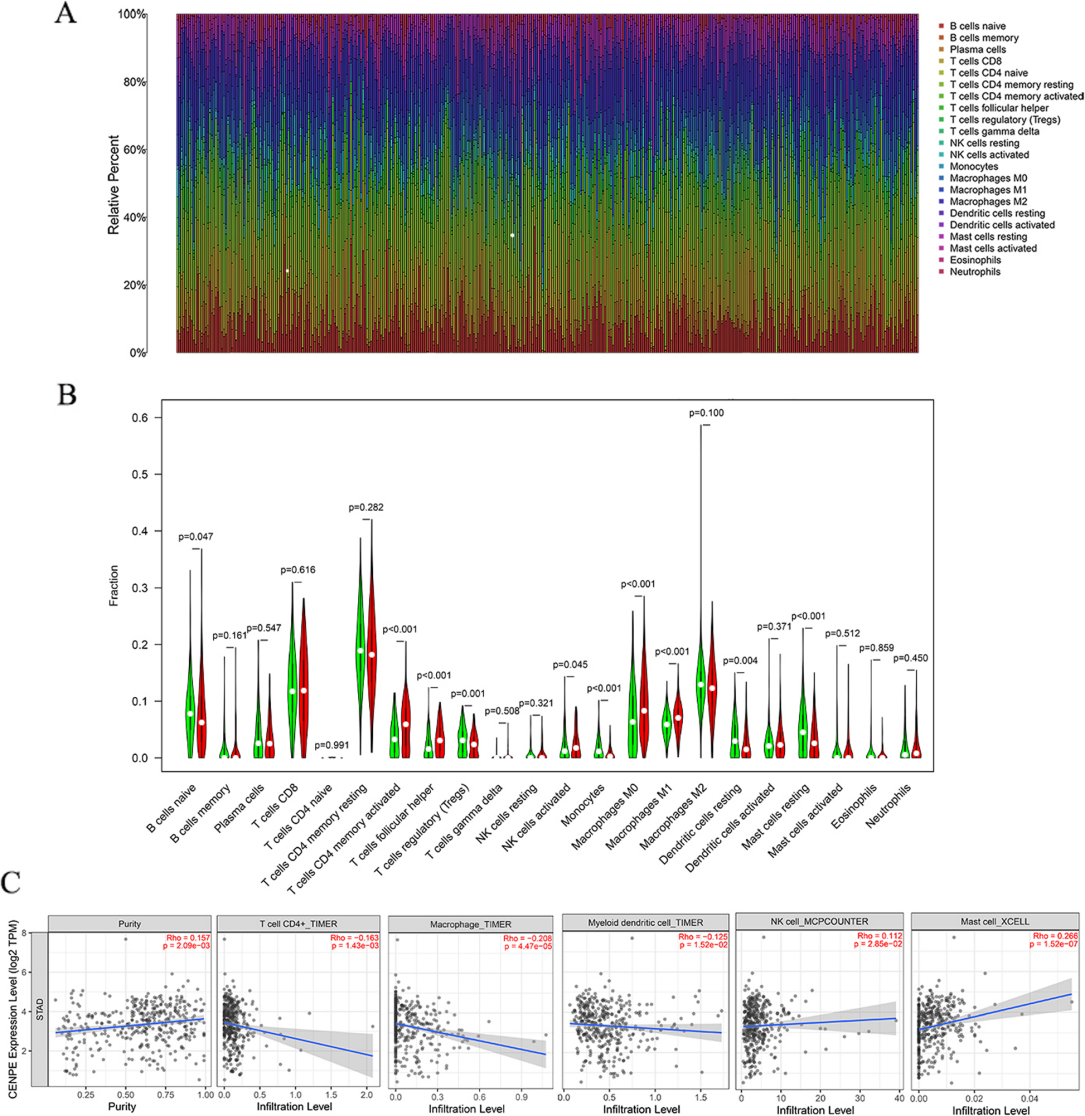
**A** Mapping of 22 immune cells in gastric cancer tissues. **B** Difference between CENPE high expression group and low expression group in 22 immune cells. **C** Correlation between CENPE expression and immune cells

Although tumor cells play a key role in tumor tissues, immune cells have also a significant influence on cancer progression. Therefore, we evaluated the association with CENPE and the tumor microenvironment, processed the expression profiles of TCGA by the CIBERSORT algorithm, analyzed the immune cells of each GC sample, and mapped the expression profiles of 22 different immune cells (Fig. 8A). Taking the median value of CENPE expression, the tumor samples of GC were separated into low and high expression groups, and the differences in the degrees of expressiveness of 22 immune cells were assessed using the vioplot package. The low-level expression group is represented by the color green, while the highly expressed group is represented by the color red. The differential analysis's findings showed that CENPE expression was connected to 10 immune cells, which included B cells naive, T cells CD4 memory activated, T cells regulatory, T cells follicular helper, NK cells activated, monocytes, macrophage M0, macrophage M1, mast cells resting, and dendritic cells resting (Fig. 8B). The relation between immune cells and CENPE expression was analyzed using Results demonstrated that CENPE expression had a negative relationship with CD4+ T cells, dendritic cells, and macrophages, which positively correlated with NK cells and mast cells (Fig. 8C).

## Discussion

Gastric cancer remains an aggressive and poorly understood malignant tumor and is the third reason for cancer deaths globally with heterogeneous presentation and tumor biology [9]. It is estimated that 732, 000 deaths occur annually, of which more than 90% of gastric cancers are gastric adenocarcinomas [9]. With the exploration and research on gastric cancer, the incidence of GC has declined, but the five-year survival rate is still poor about 10%, mainly because most gastric cancer patients have no clinical symptoms until early or even advanced stages [10]. The early detection rate for GC is 50% in wealthy nations like Japan, while the early diagnosis rate of gastric cancer in China does not exceed 10% [11]. For now, endoscopy may be a common early screening method. The development and process of GC involve many factors, such as sex, environment, H. pylori infection, and familial inheritance, which is a rather complex process. Therefore, we need to find more biological markers with specificity and high sensitivity, which are essential for the diagnosis and prognosis of GC.

CENPE is a high molecular weight kinesin (molecular weight 312 kD), one of the proteins of the spindle checkpoint, which moves unidirectionally along the microtubule track and is involved in intracellular transport and many processes of cell division [12]. It is crucial for chromosome alignment and kinetochore-microtubule attachment in cytokinesis [13]. CENPE has a kinesin motor domain at its N-terminus and its C-terminus contains two microtubule domains [14]. Interference with CENPE significantly affects chromosome motility, with CENPE inactivation leading to mitotic arrest, bringing uni-oriented chromosomes close to the spindle pole or bi-oriented chromosomes failing to align at the midblock plate [15]. Many investigations have shown that the link between and the development of several cancers, which are overexpressed in tumor cells and promote tumor cell proliferation.

Through a comprehensive analysis of online databases (TCGA and GEO), we screened GC samples and normal samples to identify the relevant genes. Our findings show that the expression of CENPE had a significant impact on the prognosis of GC patients and was closely correlated with OS (*p*< 0.05). We further demonstrate the significant expression of CENPE in gastric cancer using real-time fluorescence quantitative PCR and immunohistological analysis, indicating that CENPE may promote the development of GC. We also discovered that CENPE expression was associated with the tumor grade and the infection status of H. pylori. It is well known that stomach cancer is the result of a variety of factors, one of the most important of which is H. pylori infection. Helicobacter pylori is a pathogenic bacterium in digestive tract diseases with a spiral shape [16] and colonizes human gastric mucosa for a long time. Helicobacter pylori infection is determined by the interaction between host genetics, environment and virulence factors of the infected strain [17]. It has been found that the bacteria cause cancer mainly through specific virulence factors, including cytotoxin-associated gene A (CagA), vacuolating cytotoxin A (vacuolating cytotoxin A), VacA) and different types of outer membrane proteins. The release of these virulence factors activates relevant cellular signaling pathways, such as JAK/STAT, PI3K/Akt, ERK, Ras, and Raf [18, 19].

The results of GO and KEGG analysis showed that CENPE was related to organelle division, mitotic cell cycle phase transition, and extracellular matrix structure. Errors in mitosis cause changes in the number of chromosomes and alter the balance between chromosome numbers, leading to DNA damage, which is a driving factor in the development of tumors [20]. The extracellular matrix (ECM) provides biochemical and structural support for different cells and has been proven to regulate the progression of various cancers [21]. Studies have shown that the remodeling process of ECM in cancer cells, stromal cells, and immune cells can promote the proliferation, survival, and spread of tumor cells [22]. GSEA enrichment analysis showed that CENPE in the high expression group was mainly concentrated in amino acid TRNA biosynthesis, base excision repair, cell cycle, nucleic acid excision repair, egg meiosis, pyrimidine metabolism, RNA degradation, and spliceosomal pathway.

We found that CENPE was negatively correlated with CD4+ T cells, dendritic cells, and macrophages, and positively correlated with NK cells and mast cells. Mast cells are innate immune cells in the tumor microenvironment, which mainly rely on the release of cytokines and growth factors in the regulation of tumor growth [23]. Mast cell density is associated with the prognosis of cancer patients [24, 25]. In Liu et al. 's study, mast cells were found to promote the progression of colorectal cancer and may become a new target for immunotherapy treatment of cancer in the future [26].

It is important to acknowledge that our study has certain limitations and shortcomings. Firstly, some of the data we used were sourced from public databases, which may have affected the accuracy and quality of our results. Secondly, there could be errors in the collection of clinical samples. Lastly, the mechanism of action for CENPE in GC is not yet fully clean and will require further exploration in future research.

## Conclusions

Our study confirms that CENPE is highly expressed in gastric cancer and correlates with the prognosis of gastric cancer patients, which provides a basis for the diagnosis and prognosis of gastric cancer.

## Data Availability

The raw data could be obtained from online databases including the TCGA database (https://portal.gdc.cancer.gov/), the GEO database(https://www.ncbi.nlm.nih.gov/geo/), GEPIA (http://gepia.cancer-pku.cn/), GESA(https://www.gsea-msigdb.org/gsea/login.jsp), UALCAN (https://ualcan.path.uab.edu/index.html), the TIMER2.0 database (http://timer.cistrome.org/) without any restrictions. R code and R data files for analysis are also available if required.
